# Knowledge of Use of Antibiotic, its Resistance and Consequences among Students in Private Schools

**DOI:** 10.31729/jnma.3672

**Published:** 2018-08-31

**Authors:** Satish Kumar Deo, Sita Rijal, Sita Devi Kunwar, Anuja Dahal, Sujaya Gupta

**Affiliations:** 1 Department of Clinical Pharmacology, Institute of Medicine, Maharajgunj, Kathmandu, Nepal; 2 Om Health Campus Private Ltd., Chabahil, Kathmandu, Nepal; 3 Department of Pharmacy, Institute of Medicine, Maharajgunj, Kathmandu, Nepal; 4 Deparment of Periodontics, Kantipur Dental College, Basundhara, Kathmandu, Nepal

**Keywords:** *antibiotic resistance*, *antibiotic use*, *knowledge*, *Nepal*, *students*

## Abstract

**Introduction:**

Self-medication among adolescents has become a serious global problem that plays an important role in irrational use of medication and tends to increase with the age of adolescents. Limited research has been done in Nepal regarding antibiotic knowledge among non-medical students. Hence, this study aims to assess the knowledge of higher secondary non-science students regarding antibiotic use, resistance and its consequences.

**Methods:**

A cross-sectional descriptive study was done during antibiotic awareness week 2017 among 471 grade 11 and 12 non-science students of private schools in Kathmandu. Convenience sampling technique was used. Data was collected through self-administered questionnaires. Descriptive statistics was utilised to find out the knowledge level of the students.

**Results:**

The mean age of the students 245 (52%) male, 226 (48%) female) was 17.19 years and 335 (71.1%) were from grade 12. Approximately all 462 (98.1%) the participants had inadequate knowledge regarding antibiotic and their knowledge mean score was 5.8±2.56. More than half 277 (58.8%) of the students had not heard about antibiotic resistance, among those who had heard 113 (24%) said that doctors and nurses were the source of information.

**Conclusions:**

Almost all of the participating students lacked adequate knowledge regarding antibiotic. Thus, it is imperative to create school and community based awareness programs by policy makers for adolescents to upgrade their knowledge on safe use of antibiotics as well as to prevent the consequences of antibiotic resistance during their adult life.

## INTRODUCTION

Self-medication among adolescents has become a serious problem. It plays a significant role in the irrational use of medication and tends to increase with age.^1,2^ Antibiotic resistance is accelerated by the misuse and overuse of antibiotics.^3^ If appropriate steps are not taken immediately, the arrival of post-antibiotic era is inevitable, when the patients will die even with the minor infections.

Since its commencement, the antimicrobial resistance (AMR) surveillance programs in Nepal have not been up to the mark.^4^ Few research has been done in Nepal about antibiotic awareness among non-medical students. The lack of basic knowledge among public, stimulates irrational demand for antimicrobials and/or treatment non-adherence, which are two key factors of AMR.^5^

The study findings would be an important reference for determining the scope of the problem and helpful for formulating school and community-based awareness programs regarding antibiotic use and resistance.

The purpose of this study was to assess knowledge of higher secondary ( + 2) students regarding antibiotic use and its consequences.

## METHODS

This is a cross-sectional descriptive study conducted among five of 185 private higher secondary schools of Kathmandu valley. The five schools were selected on the basis of support from the school administration. All grade 11 and 12 students of non-science (Humanities and Management) group from each school were selected for this study. Convenience sampling technique was used to select private schools and students. Sample size was calculated by assuming that the knowledge of students should be about 50% as it has been found after pre-testing the tool in 10% study population and also from literature review. Considering possible decrease response rate researcher increased the sample size by 10%. Total Sample size of this study= 384 + 10% of 384= 422. Since all grade 11 and 12 students of non-science group who were present on the data collection day were selected for this study. The sample size of this study was 471 students. Semi-structured questionnaire was developed after reviewing the related literature to obtain the information regarding knowledge of antibiotic use, its resistance and consequences and questions were divided into two parts as semi-structured questions related to the socio-demographic variables and structured questions related to the knowledge of antibiotic use, its resistance and consequences. The adequacy and accuracy of content of the instrument was established by designing the questionnaire based on the study objectives, taking help from the previous literature and studies, consulting experts and discussing with colleagues. Instrument was formed in English language and opinion of the language expert was obtained for comprehensibility and simplicity of language. Written permission was taken from concerned private higher secondary schools before the study. The schedule for data collection was prepared according to the suitable date and time (after lunch 1 pm) by consulting each school's administration. The students of grade 11 and 12 of each school were kept separately in their own classrooms. Informed verbal consent was taken from students before data collection. The students were assured of the confidentiality of the information given by them. The students were told that they were allowed to refuse to participate in the study at any time if they wish. Then the self-administered questionnaires were distributed to the students. It took five days to collect data from five schools as each day was allocated for one school. The collected data was coded and entered in computer software package (SPSS 21.0) program. The data analysis was done by using descriptive statistics. Findings were interpreted through percentage and frequency tables to show the students' knowledge level.

## RESULTS

Out of 471 students the mean age was 17.19 years, 247 (52.4%) were male and 224 (47.6) were female and 136 (28.9%) from grade 11 and 335 (71.1 %) from grade 12.

There were eight questions related to knowledge with the total score of 24, scoring as 1 for correct response and 0 for the incorrect response (Table 1 and 2). Inadequate and adequate knowledge was defined as a total knowledge score of 0–12 and 13–24, respectively. The mean knowledge score in this study finding was 5.8±2.56 (Table 3 and 4).

**Table 1:** Knowledge regarding antibiotics.

		n (%)
Antibiotic kills bacteria	Correct	238 (50.5)
	Incorrect	233 (49.5)
Use of antibiotics^*^	Fever	231 (49.1)
	Aches and pains	125 (26.6)
	Cough^†^	123 (26.2)
	Skin wounds^†^	117 (24.9)
	Diarrhea^†^	113 (24.0)
	Sore throat^†^	100 (21.3)
	Runny nose	98 (20.9)
	Nasal congestion	63 (13.4)
	Vomiting	54 (11.5)
Way of taking antibiotic^*^	Water^†^	437 (93.0)
	Juice	18 (3.8)
	Tea	5 (1.1)
	Other methods	23 (4.9)
Side effects of antibiotics^*^	Headache^†^	220 (47.1)
	Vomiting^†^	143 (30.6)
	Nausea^†^	128 (27.4)
	Drug resistance^†^	85 (18.2)
	Abdominal pain^†^	65 (13.9)
	Rash^†^	37 (7.9)
	Diarrhea^†^	25 (5.4)
	Don't know	49 (10.5)
When to stop taking antibiotic	When you have taken all antibiotics as directed^†^	221 (46.9)
	When you feel better	192 (40.8)
	Don't know	58 (12.3)

* *multiple response*

† *correct answer*

**Table 2:** Source of information regarding antibiotic use and its resistance.

		n (%)
Source of information^*^	Doctor / nurse	68 (24.7)
	Family/friends	60 (21.8)
	Media	55 (20.0)
	Can't remember	55 (20.0)
	Pharmacist	48 (17.5)

* *multiple response

**Table 3:** Knowledge regarding antibiotic resistance.

		n (%)
Heard about antibiotic resistance	Yes	194 (41.2)
	No	277 (58.8)
Meaning of antibiotic resistance^*^	Using antibiotic when they are not necessary^†^	92 (32.7)
	Using antibiotic without physician prescription^†^	64 (22.8)
	Not completing the full course of antibiotic^†^	49 (17.4)
	Using antibiotic in febrile illness^†^	46 (16.4)
	Using the same antibiotic with different brand^†^	26 (9.3)
	Taking antibiotic with another drug^†^	25 (8.9)
Consequences of antibiotic resistance^*^	May need more expensive medicine^†^	38 (13.4)
	May be sick for longer^†^	42 (14.8)
	May have to visit doctor more^†^	52 (18.4)
	Don't know	47 (16.6)

* *multiple response*

† *correct answer*

**Table 4:** Knowledge level of students.

		n (%)
Knowledge level	Adequate	9 (1.9)
	Inadequate	462 (98.1)
Knowledge mean score	Minimum score	1
	Maximum score	21
5.85±2.56		

## DISCUSSION

In the present study nearly all (98.1%) students had inadequate knowledge regarding antibiotic that is similar with the study findings from Nigeria^6^ and survey done in India^7^ where overall antibiotic knowledge was poor. The mean knowledge score in this study was 5.8±2.5 which is different from that done in Canada where the mean knowledge score was 10.3±3.6.^8^

Half (50.5%) of the students in this study gave correct response by saying that antibiotic kills bacteria. This is in contrast to majority respondents of Poland (80%),^9^ Canada where (81.4%),^8^ and China (81.81%)^10^ respectively who knew that antibiotic kills bacteria. Regarding the response on when to stop taking antibiotic, 40.8% answered incorrectly as they replied after feeling better which is higher than the results from 12 World Health Organization (WHO) member states (32%)^11^ and Italy (15%).^12^

In response to the use of antibiotics 49.1%, 26.2% and 21.3% said that it is for fever, cough and sore throat respectively which is lower than findings from three studies in Canada 55% said for sore throat and 30% for treating common cold and cough,^8^ Nepal (94.1%) told for fever, 90.7% for sore throat and 83.1% for cough^13^ and Jordan 67.1% believed that antibiotics treat common colds.^14^

Regarding the knowledge on side effect of antibiotic 36.6% students said vomiting which is higher than the findings from Nepal where 26.2% of the respondents had mentioned nausea vomiting as a major side effect.^13^ On the other hand, these findings are contradictory with the study findings from Pakistan, where sleep disturbance was the most commonly known (46.5%) side effect.^15^ Only 21.5% students answered the questions regarding side effects correctly in this study which is lower than the study results of Swedish population (<70%).^16^

Forty-one percent of the students had heard about antibiotic resistance which is lower than the findings from a study in Canada (86%)^8^ but higher than Pakistan (20.9%).^15^ Out of those who have heard, 24% said that doctors and nurses were the source of information about antibiotic use and its resistance. This -43 ■ finding is lower than two study findings from Ecuador where the majority in both groups (56% of adolescent mothers and 68% of the adult mothers) ^17^ and Poland 72% (73% in rural and 70% in urban) indicated that their knowledge about antibiotics came primarily from their physicians and medical centers.^18^ More than half (59%) of the students in this study finding had not heard about antibiotic resistance which is lower than the findings from a study in Nepal (70.8%).^13^ Nearly one-third (32.7%) students knew that antibiotic resistance occurs by using antibiotic when they are not necessary. This is higher than the study finding from Pakistan (19.9%).^15^ The varying responses in different countries could be because of differing education systems, cultural practices, existing laws governing antibiotic prescription and usage and interventions from authorities.

There are few limitations in this study. Convenience sampling technique was used but in order to increase generalization larger scale sample size was taken by selecting five private higher secondary schools to ensure diversity among participants. Assessment of the knowledge was done through self-report so accuracy can be affected by the students' characteristics and their physical condition.

## CONCLUSIONS

Nearly all of the participating students lacked adequate knowledge about antibiotic. Thus it is imperative to create school and community-based awareness program by policy makers for adolescents to upgrade their knowledge on safer use of antibiotics as well as to prevent the consequences of antibiotic resistance during their adult life.
